# 1704. Epetraborole, a Novel Bacterial Leucyl-tRNA Synthetase Inhibitor, Demonstrates Potent Efficacy and Improves Efficacy of Standard of Care Regimen Against *Mycobacterium avium* complex in a Chronic Mouse Lung Infection Model

**DOI:** 10.1093/ofid/ofac492.1334

**Published:** 2022-12-15

**Authors:** Kavita De, Michelle S DeStefano, Carolyn Shoen, Michael H Cynamon, M R K Alley

**Affiliations:** Colorado State University, Fort Collins, California; Veteran's Health Research Institute, Syracuse, New York; Veteran’s Health Research Institute, Syracuse, California; Veteran’s Health Research Institute, Syracuse, California; AN2 Therapeutics, Menlo Park, California

## Abstract

**Background:**

Epetraborole (EBO) is a boron-containing oral inhibitor of bacterial leucyl-tRNA synthetase, an essential enzyme in protein synthesis; EBO demonstrates potent activity against nontuberculous mycobacteria. These studies evaluated oral doses (PO) of EBO against 5 *M. avium* complex (MAC) strains in a chronic mouse infection model either as monotherapy or in combination with standard of care [SOC; clarithromycin (CLR), rifabutin (RFB), ethambutol (EMB)].

**Methods:**

A pilot chronic efficacy study against *M. avium* 2285R evaluated EBO at 1, 10, 30, 100, 300 and 500 mg/kg PO once daily (QD) compared to 250 mg/kg CLR PO QD. C57BL/6 mice were infected with a pulmonary aerosol of 1x10^11^ CFU. Treatment was administered for 56 days starting on day 28 post-infection. The bacterial burden (CFU) in lungs was evaluated on days 1, 28 and 84 post-infection by plating serial dilutions of homogenates on Middlebrook 7H11 charcoal agar plates. An additional 4 strains of MAC were evaluated with EBO doses of 100, 200, 300 or 400 mg/kg QD compared with the SOC therapy for MAC (CLR 250 mg/kg, RFB 100 mg/kg, EMB 100 mg/kg) QD and SOC plus EBO 200mg/kg QD. Oral exposures of EBO were determined in a group of uninfected mice (**Table 1).**

**Table 1:**
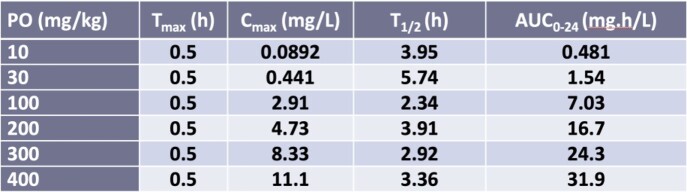
C57BL/6 Murine Pharmacokinetic Parameters

**Results:**

In a study with *M. avium* 2285R, a biofilm-forming strain, EBO at all doses tested was significantly better than CLR dosed at 250 mg/kg (**Figure 1**), and no CFU were detected on agar plates containing EBO (16 mg/L). In subsequent studies, SOC was compared to EBO in 4 additional MAC strains (**Figure 2**). Efficacy of EBO monotherapy was better than SOC against *M. avium* ATCC 700898, while it was as good as SOC with *M. intracellulare* 1956, *M. intracellulare*DNA00055, and *M. intracellulare* DNA00111 with CFU reductions ranging from 2 - 4.8 log_10_ compared to day 28 controls. In all four strains tested, 200 mg/kg EBO, which approximates the human oral equivalent dose of 500 mg, combined with SOC increased bacterial killing from 1.4 - 3.0 log_10_ CFU compared to SOC alone resulting in total lung CFU reductions of 4.6 - 5.6 log_10_.

**Figure 1:**
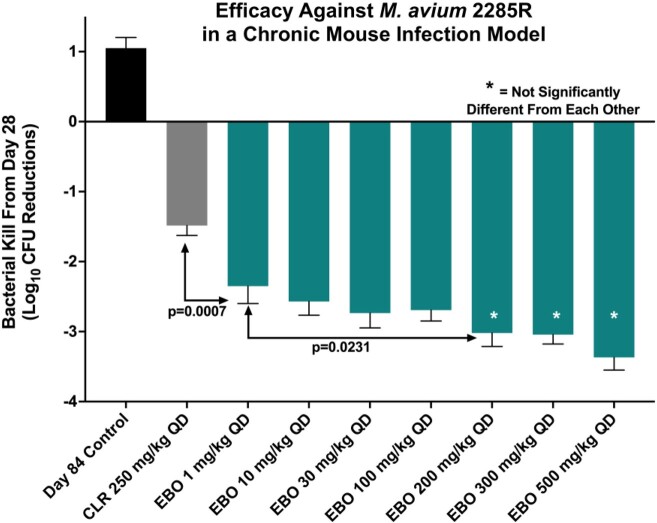

**Figure 2:**
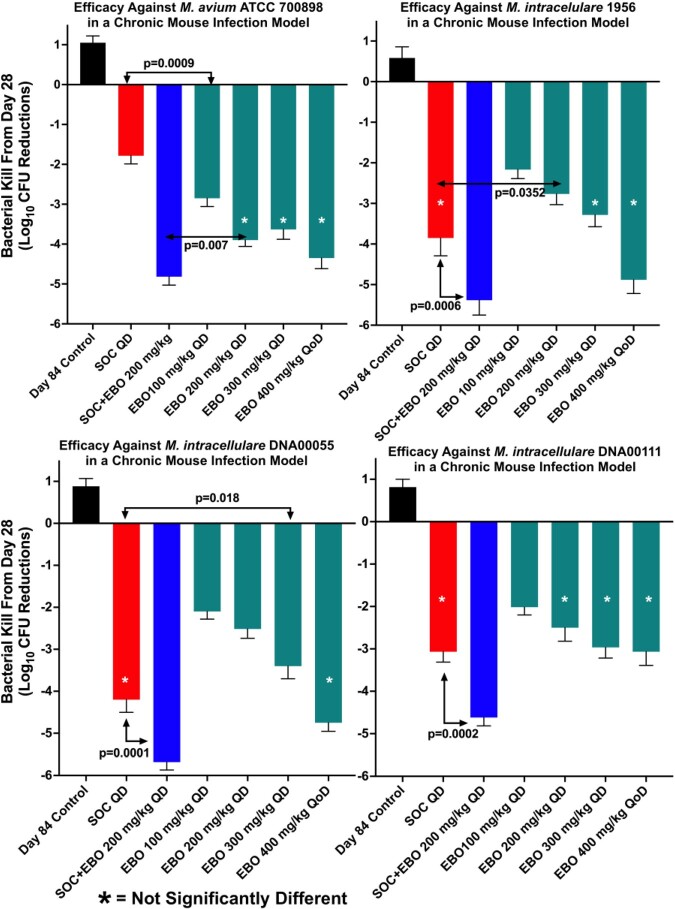

**Conclusion:**

In this chronic mouse lung infection model, no EBO resistance development was detected with *M. avium* 2285R at day 84. EBO demonstrated potent *in vivo* efficacy against 5 MAC strains and significantly improved efficacy when combined with SOC, supporting further clinical development for EBO.

**Disclosures:**

**Michelle S. DeStefano, n/a**, AN2 Therapeutics: Grant/Research Support **Carolyn Shoen, PhD**, AN2 Therapeutics: Grant/Research Support **Michael H. Cynamon, MD**, AN2: Grant/Research Support|AN2: Grant/Research Support **MRK Alley, PhD**, ABBOTT LABS: Stocks/Bonds|ABBVIE: Stocks/Bonds|AN2 Therapeutics: Author on epetraborole patent|AN2 Therapeutics: Salary|AN2 Therapeutics: Ownership Interest|AVANOS MED INC: Stocks/Bonds|NABRIVA THERAPEUTICS PLC: Stocks/Bonds|NOVARTIS AG: Stocks/Bonds.

